# A DasA family sugar binding protein Ste2 links nutrient and oxidative stress to exopolysaccharides production in *Streptomyces* sp. 139

**DOI:** 10.1186/s12866-022-02472-7

**Published:** 2022-03-08

**Authors:** Mengxin Geng, Limei Ai, Ming Ma, Panpan Li, Lianhong Guo, Guangzhi Shan, Liping Bai

**Affiliations:** 1 NHC Key Laboratory of Biotechnology of Antibiotics, CAMS Key Laboratory of Synthetic Biology for Drug Innovation, Institute of Medicinal Biotechnology, Chinese Academy of Medical Sciences & Peking Union Medical College, Beijing, 100050 China; 2 Department of Biomedical Sciences, City University of Hong Kong, Kowloon, Hong Kong People’s Republic of China; 3 Analytical & Testing Center, Institute of Medicinal Biotechnology, Chinese Academy of Medical Sciences & Peking Union Medical College, Beijing, 100050 China

**Keywords:** Ste2, DasABC, Ebosin, *Streptomyces* sp. 139, Nutrient stress, Oxidative stress

## Abstract

**Background:**

Ebosin is an exopolysaccharide produced by *Streptomyces* sp. 139, and its biosynthetic gene cluster (*ste*) has been previously described. Ste234 has high homology to the well-known ATP-binding cassette transport system DasABC, which has been linked to the regulation of morphological differentiation, antibiotics biosynthesis and aminosugars utilization in Streptomycetes. This study was conducted to evaluate the effect of the DasA family sugar binding protein Ste2 on *Streptomyces* sp. 139.

**Results:**

The disruption of *ste2* results in the upregulation of transcription of genes within Ebosin biosynthetic gene cluster and a two-fold increase in Ebosin production. RNA sequencing data suggests that the disruption of *ste2* results in the decreased utilization of carbon and nitrogen sources, increased sensitivity to oxidative stress, as well as differed strain morphology, all of which have been experimentally proven.

**Conclusions:**

Taken together, Ste2 controls Ebosin yields, aminosugars uptake, sensitivity to oxidative stress, and morphological differentiation of *Streptomyces* sp. 139.

**Supplementary Information:**

The online version contains supplementary material available at 10.1186/s12866-022-02472-7.

## Introduction

Exopolysaccharides (EPSs) are long-chain polysaccharides consisting of branched, repeating units of sugars or sugar derivatives, secreted by different microorganisms under stress conditions, and are not permanently attached to the cell surface [1]. The EPSs produced by bacteria are involved in multiple protective cellular functions that improve bacterial competition in different habitats [2]. Although a variety of bacterial EPSs have been reported, studies of EPSs from Streptomycetes remain limited. In our previous work, a novel EPS produced by *Streptomyces* sp. 139, designated Ebosin (EPS 139A), has been found to possess remarkable anti-rheumatic arthritis activity *in vivo* [3, 4]. Also, its biosynthesis gene cluster (*ste*) consisting of 27 ORFs has been identified [5–7], in which Ste234 belongs to the family of ATP-binding cassette (ABC) transporters.

ABC transporters couple ATP hydrolysis to the uptake and efflux of solutes across the cell membrane in bacteria [8]. ABC transporters can uptake a wide variety of substances including sugars, peptides, and amino acids, allowing bacteria to monitor environmental and physiological changes, i.e., nutrient depletion, and providing the way to avoid possible damages [9]. ABC transporters also function in efflux of substances, including surface components of the bacterial cell (such as capsular polysaccharides, lipopolysaccharides, and teichoic acid), proteins involved in bacterial pathogenesis (such as hemolysin, heme-binding protein, and alkaline protease), heme, drugs and siderophores [10].

Various genes encoding oligosaccharide-uptake systems have been identified in Streptomycetes: *cebEFG* for cellobiose and cellotriose in *Streptomyces reticuli* [11]; *malEFG* for maltose in *S. coelicolor* [12]; *ngcEFG* for N-acetylglucosamine (GlcNAc) and (GlcNAc)_2_ in *Streptomyces olivaceoviridis* [13]; and *bxlEFG* for xylobiose in *Streptomyces thermoviolaceus* [14]. Interestingly, all of these operons encode subunits of ABC transporters, with the *E* and *FG* genes of these uptake systems encoding sugar-binding proteins (SBPs) (CebE, MalE, NgcE and BxlE) and two putative integral membrane proteins (CebFG, MalFG, NgcFG and BxlFG), respectively [15]. Deficient in aerial mycelium and spore formation (DasRABC) was first identified in *S. griseus* as an ATP-binding cassette transport system involved in regulation of morphological differentiation in response to glucose and is one of the best studied ABC transporters in Streptomycetes [16]. Our previous study demonstrated that *ste34* in *Streptomyces* sp. 139 are homologous to the membrane spanning protein encoding genes *dasBC* [17]. The disruption of *ste34* resulted in a mutant strain with dramatically decreased production of Ebosin [17]. Meanwhile, the effect of Ste2, which has high homology to DasA from *S. coelicolor* and *S. griseus*, remains unclear. Here, we demonstrate that the sugar binding protein Ste2 controls nutrient uptake, exopolysaccharide yields, morphological differentiation, sensitivity to oxidative stress and microbial morphology of *Streptomyces* sp. 139.

## Results

### Homology analysis of Ste2

Whole genome sequence of *Streptomyces* sp. 139 is available [18]. Amino acid alignment of Ste2 with DasA has been done with Clustal Omega Software (https://www.ebi.ac.uk/Tools/msa/clustalo/) and is shown in Supplementary Fig. 1. Ste2 shows 84% identity and 91% similarity to the sugar binding protein DasA of *Streptomyces coelicolor* A3 (2) [19, 20], 34% identity and 49% similarity to DasA of *Streptomyces griseus* [16] (Supp. Figure 1A). Homology of Ste34 to DasBC has been published in our previous research [17], and the alignment is shown in Supp. Figure 1B-C. Ste3 shows 89% identity and 94% similarity to integral membrane protein DasB of *Streptomyces coelicolor* A3 (2) [19, 20], 35% identity and 58% similarity to DasB of *Streptomyces griseus* [21] (Supp. Figure 1B). Ste4 shows 87% identity and 94% similarity to integral membrane protein DasC of *Streptomyces coelicolor* A3 (2) [19, 20], 41% identity and 63% similarity to DasC of *Streptomyces griseus* [21] (Supp. Figure 1C). We hypothesize that *ste234*, adjacent to GntR family regulator *ste1*, encodes an ATP-binding cassette (ABC)-type transporter for carbohydrate uptake as DasABC of *Streptomyces coelicolor* A3 (2) and *Streptomyces griseus.*

### Strain construction and validation

**Fig. 1:**
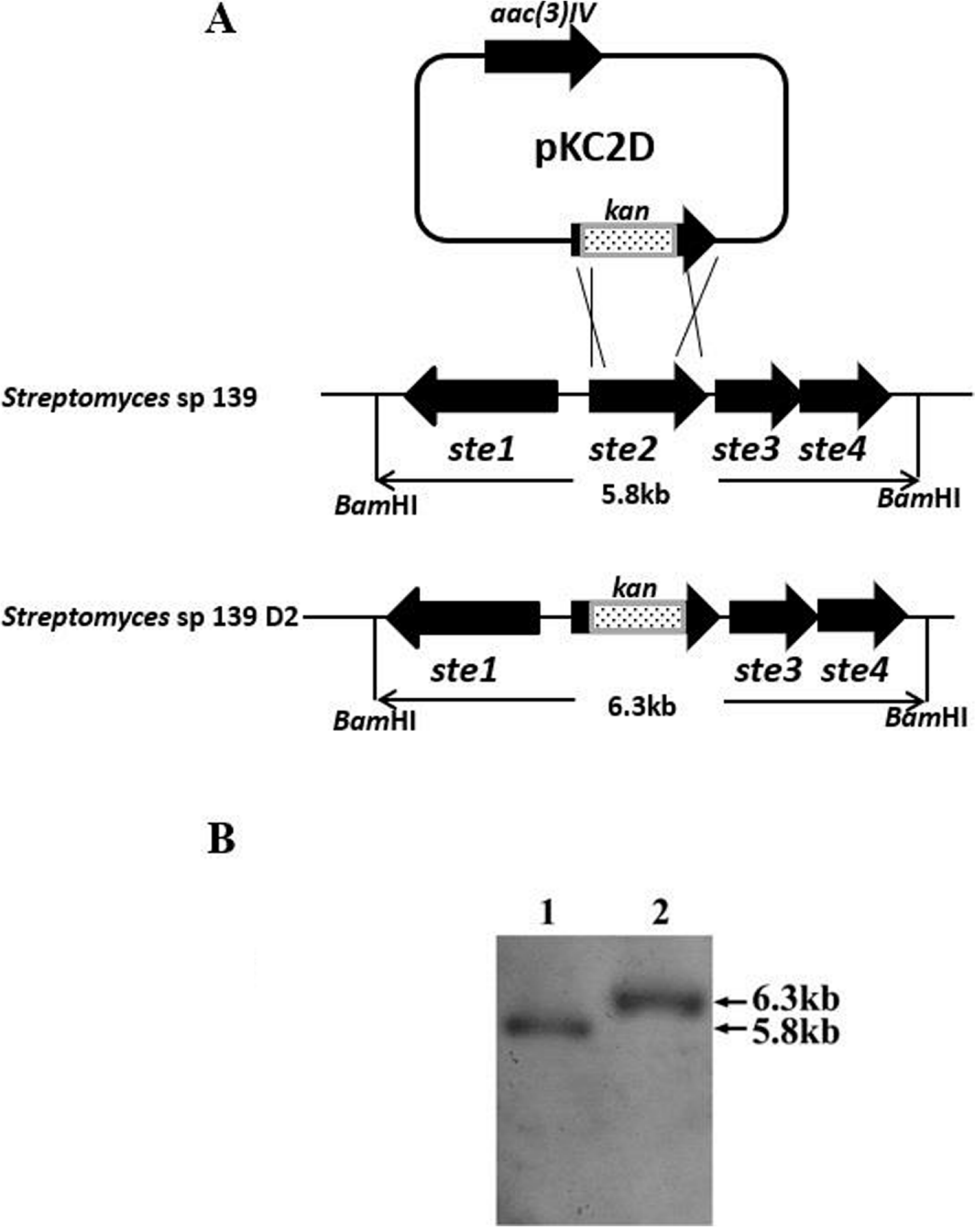
Southern blotting analyses of the wild-type *Streptomyces* sp. 139 and Strain D2. **A** Representative of the construction of the *ste2* disruption strain D2. **B** (Line 1) *Streptomyces* sp. 139 chromosome DNA digested with *Bam*HI and *Hin*dIII, (Line 2) Strain D2 chromosome DNA digested with *Bam*HI, a DIG-labeled F1 as the hybridization probe. *kan*: kanamycin resistance gene fragment

To elucidate the function of Ste2 in the biosynthesis of Ebosin, the *ste2* deletion strain (strain D2) was constructed using a double cross-over gene-replacement strategy. A number of colonies (Km^r^ Am^s^) were selected randomly and their genomic DNAs were isolated and confirmed by Southern hybridization. As shown in Fig. 1A, *ste1*-*4* is located within two *Bam*HI cut sites, giving a 5.8 kb fragment following *Bam*HI digestions. When *ste2* was replaced with the *Km* fragment, this *Bam*HI fragment was 6.3 kb (Fig. 1A). As shown in Fig. 1B, a distinctive hybridization band of 6.3 kb was detected in the *ste2*-deleted mutant strain, designated as strain D2 (Fig. 1B, line 2), and a band of 5.8 kb was obtained for wild type strain (Fig. 1B, line 1). To study whether the effects of *ste2* deletion can be reversed by the complementation of *ste2*, the complementary strain was constructed as described in the method section. Briefly, fragment *erm*E^*^ and *ste2* were ligated together and inserted into the *Bam*HI–*Hin*dIII-cut pKC1139 vector to yield pKC2C. After being propagated in *E. coli* ET12567, pKC2C was isolated and transformed into the protoplasts of strain D2. 10 transformants (Am^r^ Km^r^) were obtained and the existence of pKC2C was confirmed by restriction digestion pattern (not shown). The complementary strain was designated as strain C2.

### Disruption of *ste2* dramatically enhances Ebosin production in *Streptomyces* sp. 139

**Fig. 2:**
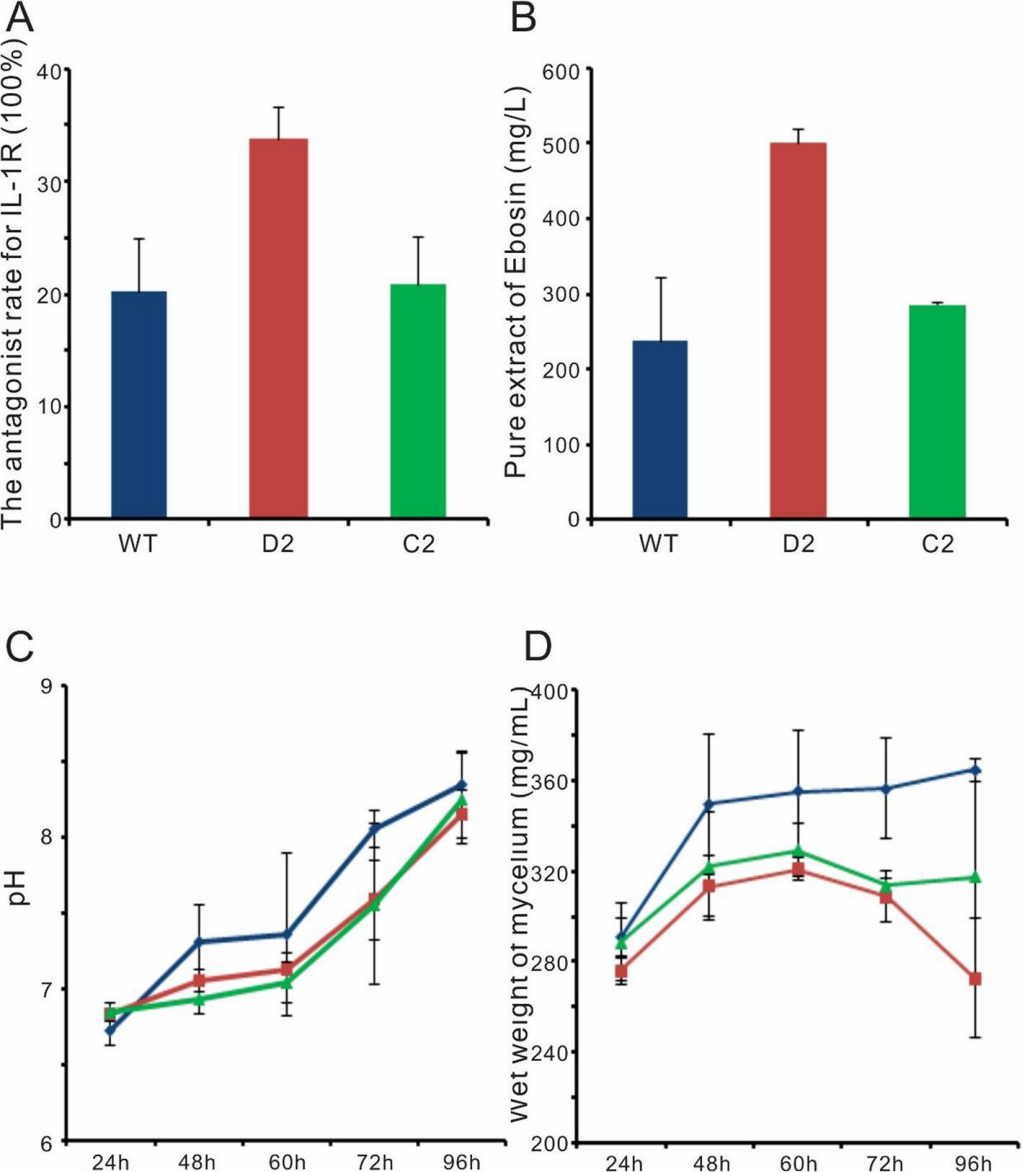
Analysis of fermentation of wild type *Streptomyces* sp. 139, mutant strain D2 and C2. Blue: wild-type strain; red: strain D2; green: strain C2. **A** The competitive binding activities of the fermentation supernatant with IL-1 for IL-1R. Samples were collected at 96 h. **B** The production of Ebosin at 96 h from *Streptomyces* sp. 139, Strain D2 and Strain C2. **C** pH of the fermentation process at different times. **D** Wet weight of mycelium of fermentation broth sampled at different time points

The fermentation supernatant at 96 h for *Streptomyces* sp. 139, Strain D2 and Strain C2 were harvested by centrifugation, and then analyzed by ELISA for the determination of its antagonist rate for IL-1R (Fig. 2A), as well as quantified for the total yield of Ebosin (Fig. 2B), respectively. The disruption of *ste2* resulted in a more than 50% increase in the supernatant’s antagonist rate for IL-1R, and the complemented strain has about the same rate as the native strain. Strain D2 has an around two-fold increase in its Ebosin production, while the complemented strain C2 has similar production level as the native strain.

The pH value of each sample was also analyzed. As the incubation time prolonged, the pH value of culture supernatant gradually increased from around 6.7 to 8.2. pH value of culture supernatant between *Streptomyces* sp. 139, strain D2 and strain C2 did not show significant difference (Fig. 2C).

The wet mycelium weight of *Streptomyces* sp. 139, strain D2 and strain C2 at each time point were shown in Fig. 2D. Mycelium density reached maximum during 48 h to 72 h incubation period, while mycelium density of *Streptomyces* sp. 139 was higher than strain D2 and strain C2.

### Expression of genes within Ebosin biosynthetic gene cluster are significantly upregulated in D2 strain

**Fig. 3:**
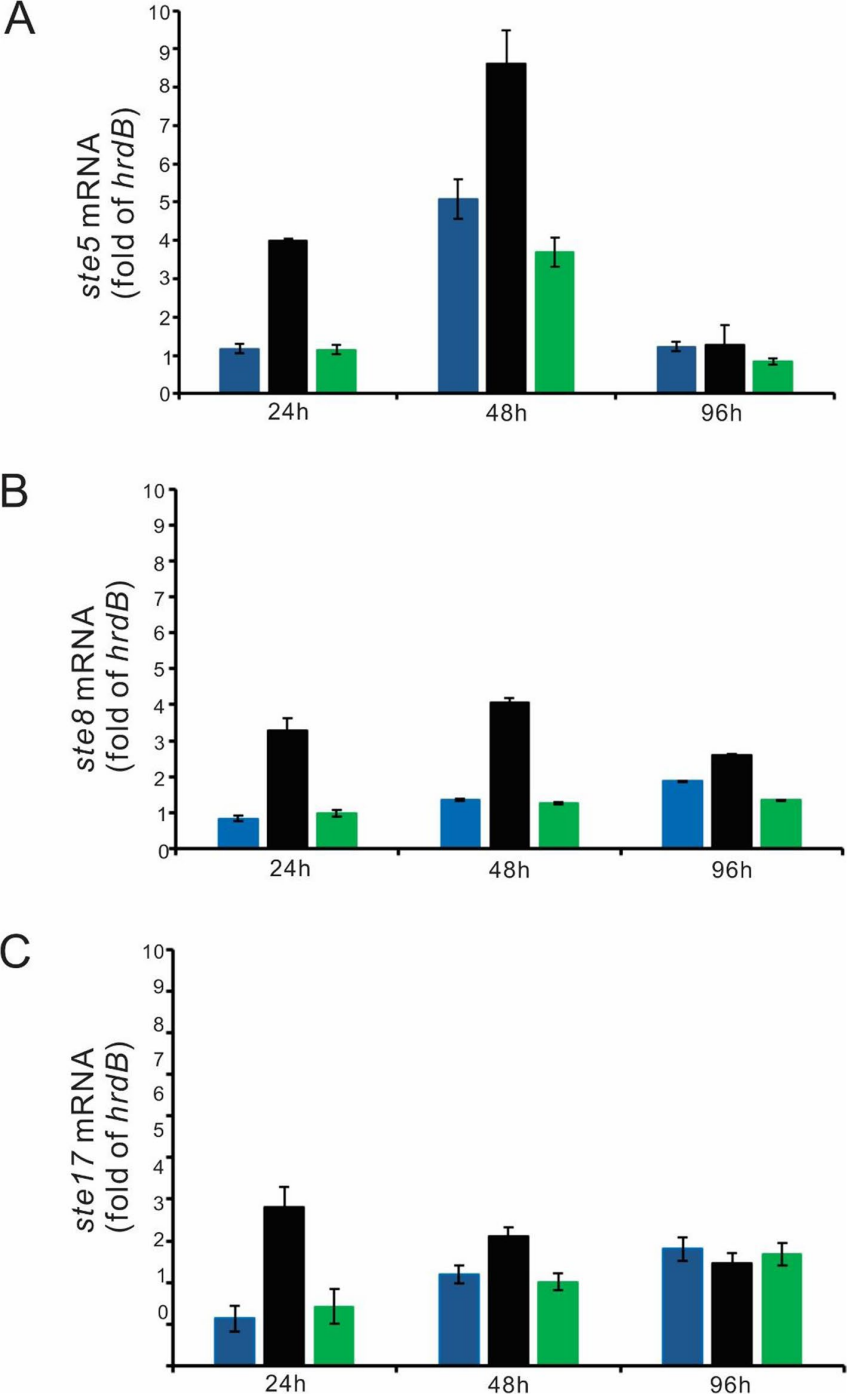
Quantification of the transcription level of *ste5* (**A**), *ste8* (**B**), and *ste17* (**C**) in *Streptomyces* sp. 139 wild-type, D2 and C2 strain. Strains were allowed to grow for 24 h, 48 h, and 96 h before analysis. The transcription level of *ste5*, *ste8*, and *ste17* was normalized to the transcription level of *hrdB*, respectively. Blue: wild-type strain; black: strain D2; green: strain C2. Experiments were done in triplicate

A RT-qPCR experiment was performed to further confirm whether genes within Ebosin biosynthetic gene cluster were regulated at certain time points (Fig. 3). The genes encoding galactosyltransferase (*ste5*) [5], chain length determinants (Wzz) (*ste8*) [5], and α-D-glucose-1-phosphate cytidylyltransferase (*ste17*) [22], respectively, were chosen for this experiment. As shown in Fig. 3, *ste5*, *ste8*, and *ste17* were upregulated for at least 3-fold in D2 strain at 24 h, all of which are statistically significant (*p* < 0.05). At 48 h, *ste5* was upregulated for around twofold in D2 strain (*p* < 0.05), *ste8* was upregulated for about threefold in D2 strain (*p* < 0.05), while the upregulation of *ste17* was not that significant (*p* > 0.05). At 96 h, there was not a significant difference in the transcription of the three genes in all strains (*p* > 0.05). The complemented strain has similar transcription level as wild-type strain at all time points (*p* > 0.05). As a conclusion, the deletion of *ste2* resulted in the upregulated transcription of *ste5*, *ste8*, and *ste17*, especially in the first 48 h, which cooperates with the fact that the Ebosin yield is promoted in D2 strain.

### Strain D2 has a defect in the utilization of GlcNAc and (GlcNAc)_2_

**Fig. 4:**
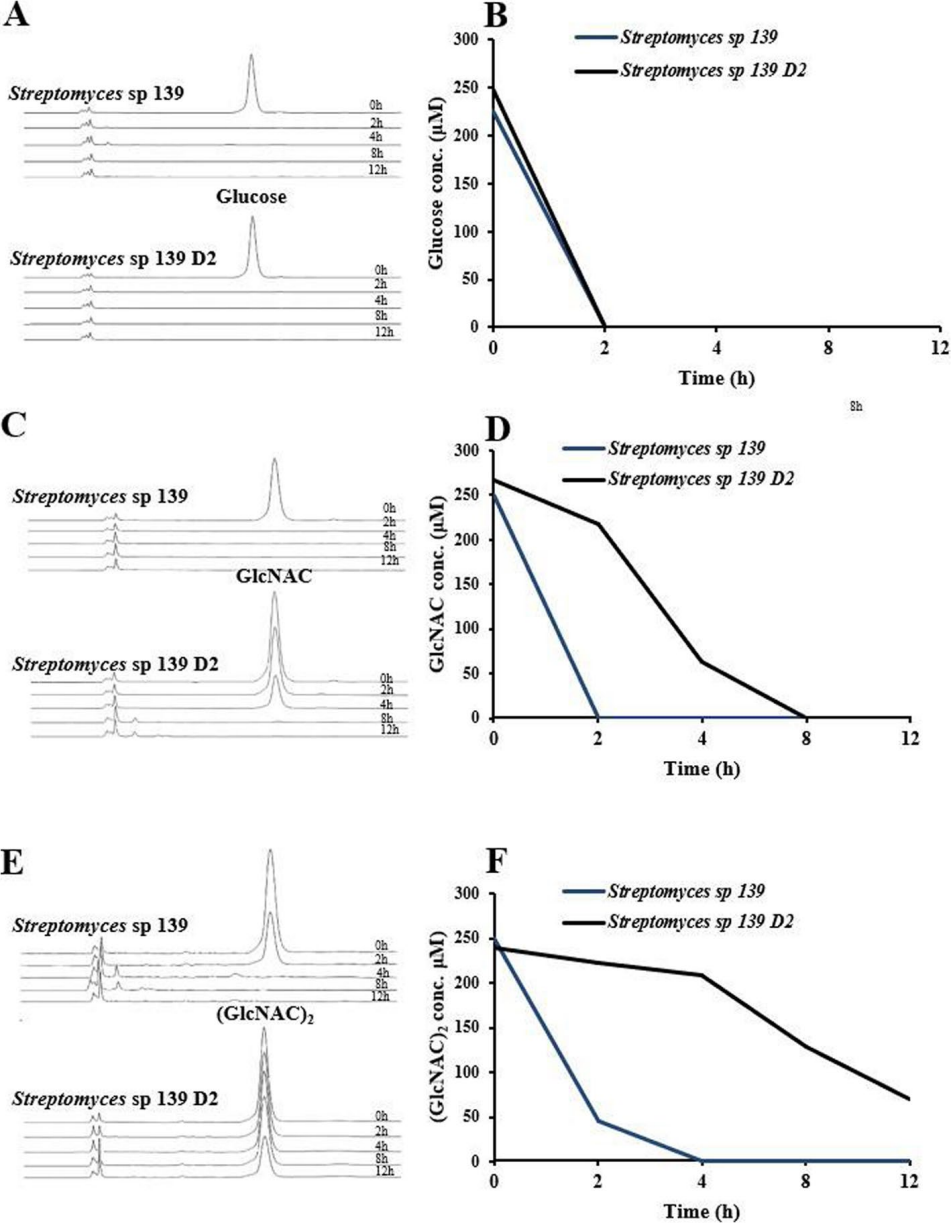
Quantification of the utilization of glucose (**A**&**B**), GlcNAc (**C**&**D**), and (GlcNAc)_2_ (**E**&**F**) in *Streptomyces* sp. 139 and mutant strain D2. S139 native and D2 strain were cultured in basic media (10 mM K_2_HPO_4_, 10 mM KH_2_PO_4_, 1 mM CaCl_2_, 0.5 mM MgCl_2_, 0.1% (v/v) trace element solution) supplemented with 250 μM of glucose (A&B), GlcNAc (C&D) and (GlcNAc)_2_ (E&F), respectively. Bacterial culture samples were taken at 0 h, 2 h, 4 h, 8 h, and 12 h, and were analyzed for the concentration of glucose, GlcNAc and (GlcNAc)_2_, respectively

To investigate whether Ste2 plays a role in the uptake of aminosugars, we quantified the rate of utilization of glucose (Fig. 4A-B), GlcNAc (Fig. 4C-D) and (GlcNAc)_2_ (Fig. 4E-F) in the supernatant of the native and D2 strain which was cultured in basic media (10 mM K_2_HPO_4_, 10 mM KH_2_PO_4_, 1 mM CaCl_2_, 0.5 mM MgCl_2_, 0.1% (v/v) trace element solution) supplemented with 250 μM of glucose, GlcNAc and (GlcNAc)_2_, respectively. Both strains absorbed all supplemented glucose within the first two hours. However, the disruption of *ste2* significantly decreased the uptake of GlcNAc and (GlcNAc)_2_. Wild-type 139 strain utilized all supplemented GlcNAc within the first 2 h, while the D2 strain utilized only 20% supplemented GlcNAc during the same period of time, and all supplemented GlcNAc at 8 h. As for (GlcNAc)_2_, wild-type strain utilized 80% within 2 h and nearly all supplemented (GlcNAc)_2_ within 4 h. The D2 strain barely utilized any (GlcNAc)_2_ within the first 4 h, around 50% until 8 h, while around 70% until 12 h. The results suggest that Ste2 plays an important role in the utilization of GlcNAc and (GlcNAc)_2_ in *Streptomyces* sp. 139.

### *ste2* null mutant has a highly aberrant phenotype

**Fig. 5:**
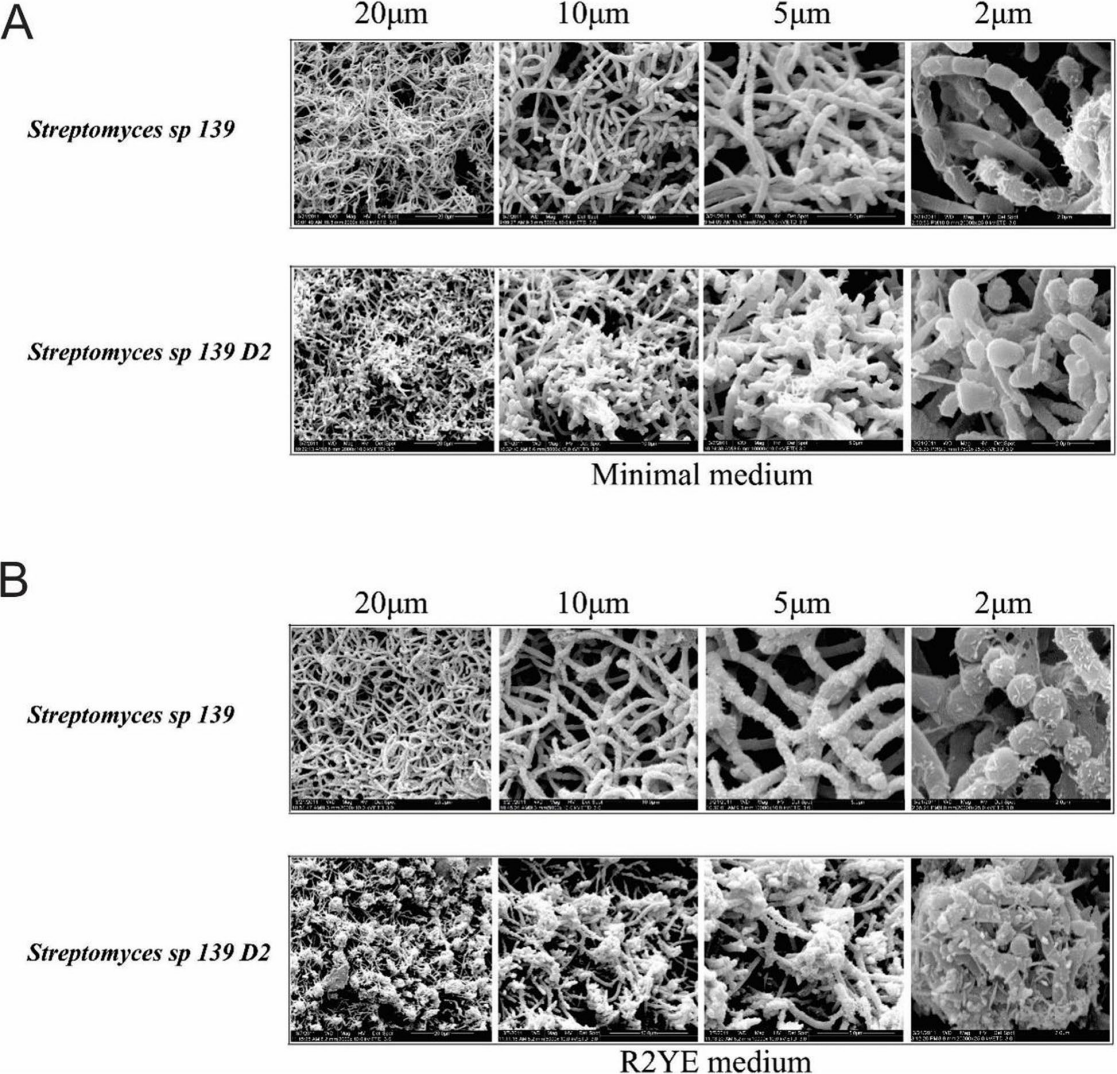
Scanning electron micrographs of Streptomyces sp. 139 and strain D2 grown on minimal agar medium (**A**) and R2YE agar medium (**B**)

A closer inspection of the native and D2 strain with a cryo-scanning electron microscope is shown in Fig. 5. On the minimum medium supplemented with glucose or R2YE agar medium, *Streptomyces* sp. 139 (WT) produced abundant and wild-type spores (Fig. 5A, B). In contrast, Strain D2 failed to produce normal aerial hyphae and spores on minimum medium supplemented with glucose (Fig. 5A). Excitingly, the *ste2* mutant on R2YE agar medium had highly aberrantly shaped spores, which produced spore heaps at a high frequency (Fig. 5B). To our knowledge, such a phenotype has not been established before in any *Streptomyces* mutant.

### Global transcriptional changes in *Streptomyces* sp. 139 D2 mutant

**Fig. 6:**
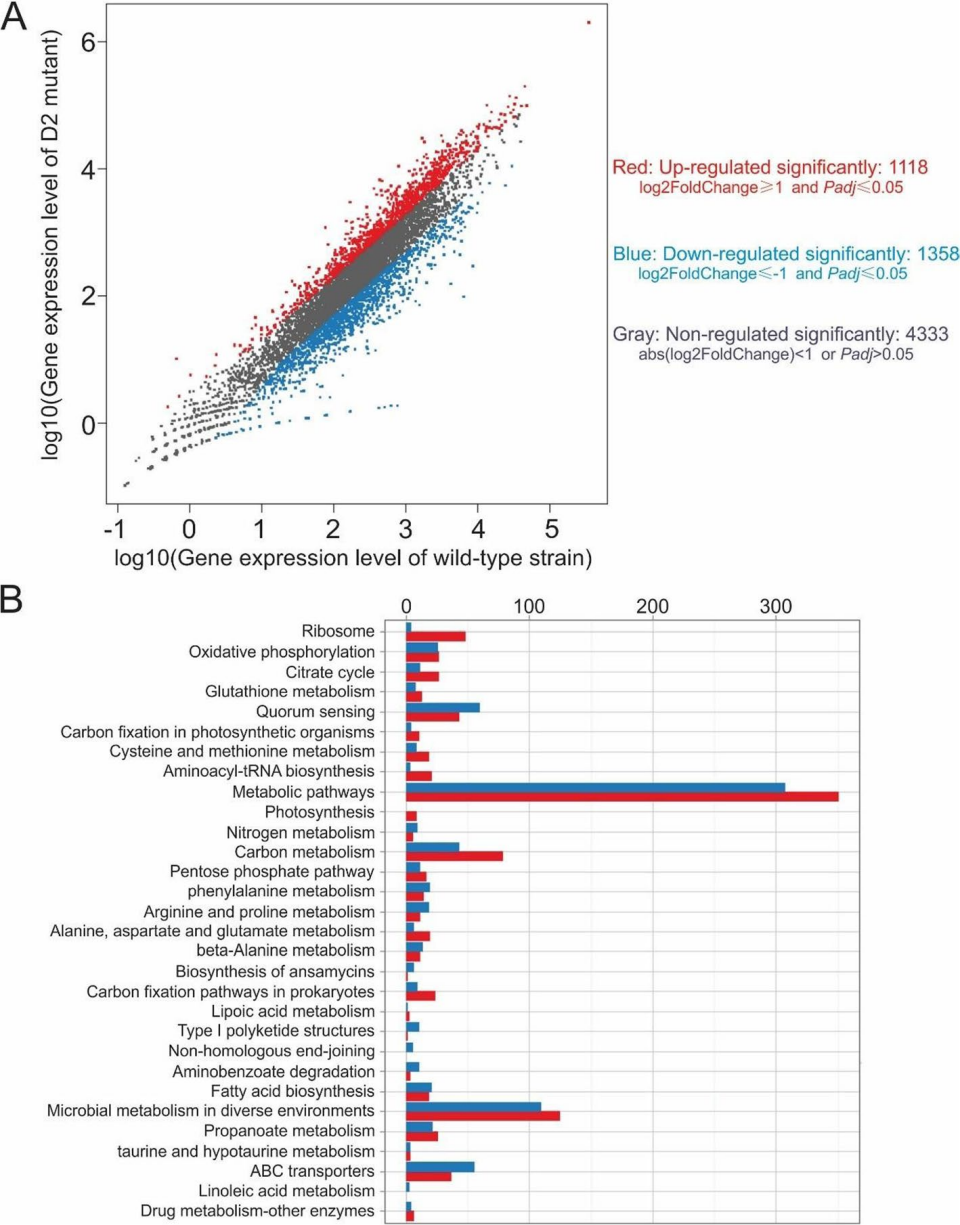
Functional categories and enrichment analysis of the DEGs**.** **A** Scatter plot of log10 gene expression levels before and after the deletion of *ste2*. **B** DEGs number of the most enriched pathway. Blue: down-regulated; Red: up-regulated

Transcriptome analysis was done in triplicate for both the wild type strain and *ste2* mutant strain. Using a cut-off of 2.0-fold difference between the wild-type and D2 mutant, 2476 genes were identified as differentially expressed. As shown in Fig. 6A, 1118 genes were upregulated and 1358 genes were downregulated for more than 2.0-fold in D2 mutant when compared with wild-type strain, and those changes are statistically significant (Adjusted P value <  = 0.001 [23]). 4333 genes were not regulated in a statistical significantly manner. According to the National Center for Biotechnology Information (NCBI) S139 genome annotation, gene-annotation enrichment and functional annotation clustering analysis of differentially expressed genes (DEGs) were conducted [23]. As shown in Fig. 6B, the DEGs were enriched in thirty significantly changed terms in Kyoto Encyclopedia of Genes and Genomes (KEGG) pathways. According to the KEGG pathways definition, most of the genes being significantly regulated are involved in metabolic pathways, following which are microbial metabolism in diverse environments, quorum sensing, carbon metabolism, ABC transporters, etc. (Fig. 6B).

According to the RNA sequencing analysis provided by Beijing Genomics Institute (Table S1), we found that 111 genes have a higher than eight-folds difference in their expression level in the *ste2* mutant strain compared with wild type strain. Within the 111 genes, 86 genes were down-regulated, while 25 genes were up-regulated. Most of the genes have a close relationship with metabolic pathways, microbial metabolism in diverse environments, quorum sensing, as well as carbon and nitrogen metabolism. It is clear that in *ste2* mutant strain, some genes with their expression levels significantly differed from wild strain are involved in carbon/nitrogen fixation and cellular response to oxidative stress. For example, a gene encoding a carbohydrate ABC transporter substrate-binding protein has a 15.7-fold increase in its expression level in *ste2* mutant strain. The protein is a periplasmic component within an ABC-type glycerol-3-phosphate transport system and is involved in carbohydrate transport and metabolism. A gene encoding an MBL fold metallo-hydrolase has an 18-fold increase in its expression level in *ste2* mutant strain. The protein is a L-ascorbate metabolism protein and is involved in carbohydrate transport and metabolism.

A selected collection of genes of interest have been summarized in Table 1. Apart from genes regulating carbon metabolism, two genes encoding methionine adenosyltransferase and glutamate-ammonia ligase have been upregulated for around three- and seven-folds, respectively. It suggests that the amino acid metabolism in *ste2* disruption strain is also promoted. Excitingly, two genes encoding putative N,N'-diacetylchitobiose transport system substrate-binding proteins have a 6.47-fold and 2.17-fold decrease in their expression levels in *ste2* mutant strain, respectively, which corroborates with the decreased uptake of GlcNAc and (GlcNAc)_2_ in the D2 strain. Interestingly, a gene encoding a cyclic-di-GMP-binding protein has a 3.7-fold increase in the D2 strain. It has been previously reported that the bacterial EPSs are regulated for c-di-GMP, and c-di-GMP also plays a key role in the utilization of nitrogen source, the biosynthesis of multiple nucleoside precursors, and response to oxygen, nitric oxide, and a variety of other environmental challenges [24, 25]. However, c-di-GMP binding proteins are highly diverse, and identification of them remains a difficult and incomplete task [26], thus the increase in the expression level of the c-di-GMP-binding protein in the D2 strain needs future investigation.

**Table 1:** Selected genes being significantly up- or down-regulated in the *ste2* disruption strain D2. The function, KEGG orthology, and a result of BLAST are shown for each gene, respectively

Function	Gene ID	D2 to WT Ratio	KEGG Orthology	Blast nr
Carbohydrate metabolism	S139GL004816	15.74661713	alpha-glucoside transport system substrate-binding protein	carbohydrate ABC transporter substrate-binding protein [*Streptomyces* sp. CNH189]
	S139GL004057	18.01236617	peptidoglycan DL-endopeptidase CwlO	MBL fold metallo-hydrolase [*Streptomyces* sp. CNH189]
Amino acid metabolism	S139GL002102	2.958184062	S-adenosylmethionine synthetase	methionine adenosyltransferase [*Streptomyces* sp. CNH189]
	S139GL005081	6.721092236	glutamine synthetase	glutamate–ammonia ligase [*Streptomyces*]
Membrane transport	S139GL002390	0.154636472	N,N'-diacetylchitobiose transport system substrate-binding protein	putative sugar transporter sugar-binding protein [*Streptomyces* sp. 139]
	S139GL002391	0.461712269	N,N'-diacetylchitobiose transport system permease protein	putative sugar transporter integral membrane protein [*Streptomyces* sp. 139]
Response to oxidative stress	S139GL004328	0.226084044	catalase-peroxidase	catalase/peroxidase HPI [*Streptomyces* sp. CB02400]
	S139GL003847	0.196900796	ATP-binding cassette, subfamily C, bacterial CydCD	ABC transporter ATP-binding protein [*Actinobacteria*]
	S139GL000959	5.552515326	peroxiredoxin [alkyl hydroperoxide reductase subunit C]	peroxiredoxin [*Streptomyces* sp. CNH189]
	S139GL001510	4.306959158	aldehyde dehydrogenase [NAD +]	aldehyde dehydrogenase [*Streptomyces* sp. CNH189]
Metabolism/ regulation	S139GL000617	3.71384308	cyclic-di-GMP-binding protein	DUF520 domain-containing protein [*Streptomyces*]
Cell morphology	S139GL003161	0.45401102	rod shape-determining protein MreB and related proteins	hypothetical protein [*Streptomyces* sp. CNH189]
	S139GL005336	0.179128887	rod shape-determining protein MreB and related proteins	rod shape-determining protein [*Actinobacteria*]
	S139GL005480	2.244936284	rod shape-determining protein MreB and related proteins	rod shape-determining protein [*Streptomyces*]

The differed morphology of wild-type and D2 strain might be attributed to the differed expression of three rod shape-determining protein MreB and related proteins, in which two genes are down-regulated, while one gene is up-regulated. MreB, the cell shape-determining bacterial actin homologue, has been demonstrated to be required for the maintenance of a rod-shaped cell and formation of spirals that traverse along the longitudinal axis of *Bacillus subtilis* and *E. coli* cells; MreB filaments also function as a cytoskeleton, serving as an organizer or tracking device for the cell wall morphogenesis in *Caulobacter crescentus* [27]. In *S. coelicolor*, MreB has been demonstrated to localize underneath the internal spore wall but not in vegetative mycelium, and has been suggested to function in the formation of environmentally stable spores [28]. The regulation of cellular shape might be a complicated process in *Streptomyces* sp. 139 strain and future work will be required.

Genes involved in responding to oxidative stress are regulated in a more complicated manner. A gene encoding a catalase-peroxidase (S139GL004328) is down-regulated for more than four-folds in D2 strain. A homologue of the catalase-peroxidase KatG in *E. coli* has been demonstrated to have both catalase and broad-spectrum peroxidase activity, plus NADH oxidase, INH lyase and isonicotinoyl-NAD synthase activity, in which INH lyase and isonicotinoyl-NAD synthase are responsible for the activation of isoniazid as an anti-tubercular drug [29]. It suggests the D2 strain will be more sensitive to oxidative stress than wild-type strain. A gene encoding a homologue to the bacterial heterodimeric ABC transporter CydCD is down-regulated for more than five-folds in D2 strain. In *E. coli*, CydCD is required for the biogenesis of both cytochrome *bd*-type quinol oxidases and periplasmic cytochromes. CydDC could also act as a thiol transporter [30]. Thus the disruption of *ste2* may also render the strain to be more sensitive to oxidative stress. In the meantime, genes encoding homologues of peroxiredoxin and aldehyde dehydrogenase in *Streptomyces* sp. CNH189 are upregulated for 4.3- and 5.6-folds in D2 strain, suggesting that the survival rate of D2 strain may be increased under oxidative stress. For more information, a full list of genes whose expression changed significantly is available in Table S1.

To summarize, we hypothesize that the deletion of *ste2* enhances the strain’s ability to metabolize carbohydrates and amino acids, to sense and deal with oxidative stress from hydrogen peroxide and aldehydes in the outside environment; in the meantime, the deletion of *ste2* decreases the strain’s ability to uptake aminosugars like N,N'-diacetylchitobiose. Morphology of the strain is also affected by differed expression of multiple rod shape-determining proteins.

### Strain D2 is more sensitive to hydrogen peroxide than the wild-type strain and strain C2

**Fig. 7:**
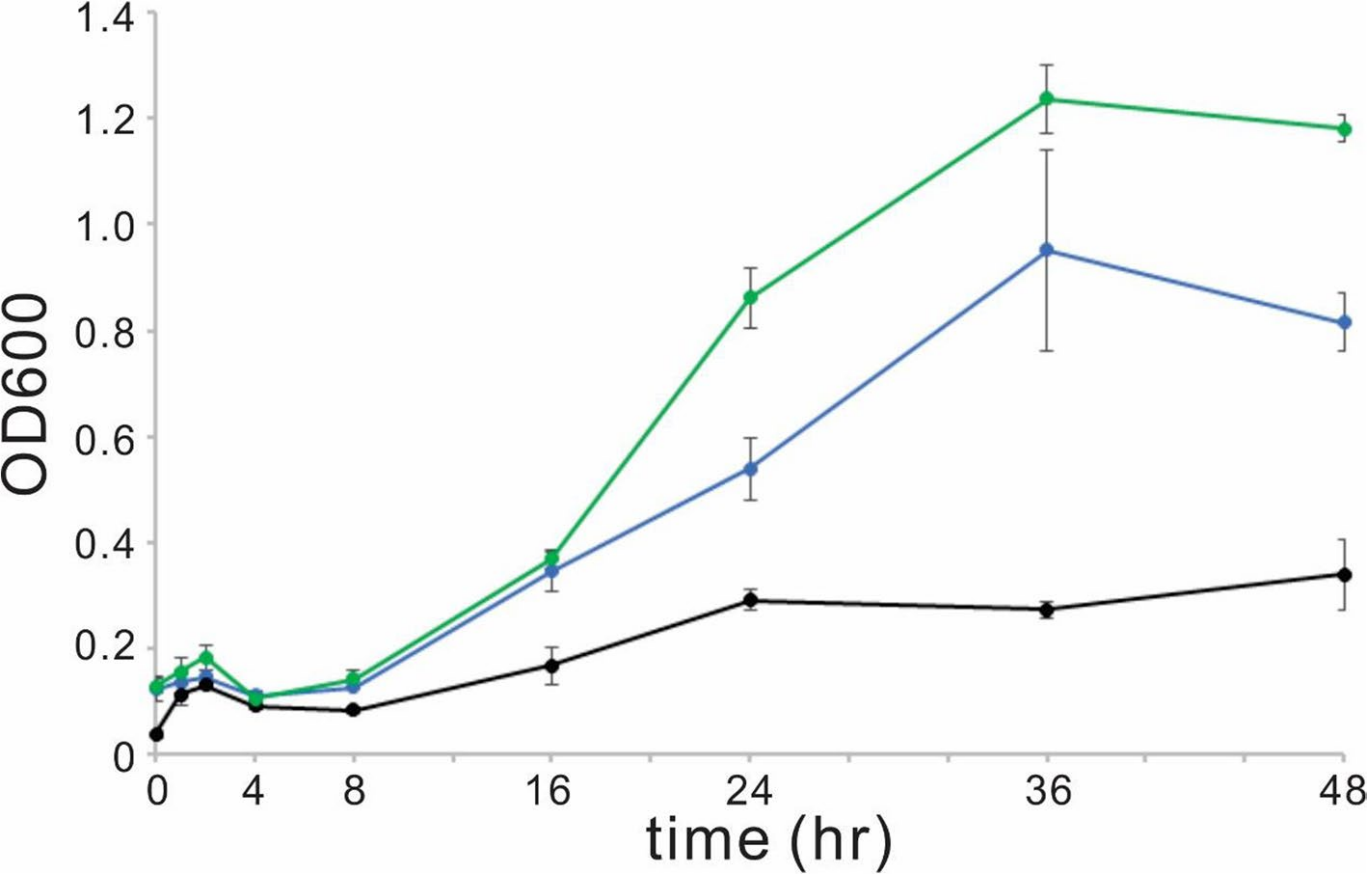
Bacterial growth under the treatment of 0.1% hydrogen peroxide. Samples were taken and analyzed for OD600 at 0, 1, 2, 4, 8, 16, 24, 36, and 48 h. Black line: strain D2; blue line: wild-type strain; green line: strain C2. Experiments were done in triplicate

Global transcriptional changes in *Streptomyces* sp. 139 D2 mutant revealed that the disruption of *ste2* may render the strain to be more sensitive to oxidative stress. We decided to test whether hydrogen peroxide has a differed effect in the growth of wild-type and D2 strain, as hydrogen peroxide is a natural source of oxidative stress. Wild-type strain and strain C2 are less sensitive to hydrogen peroxide compared with strain D2. The MIC of hydrogen peroxide against wild-type strain and strain C2 is 1%, while is 0.25% against strain D2. Thus Ste2 might play a role in protecting the strain from oxidative stress, which corroborates with the RNA seq analysis. To further investigate the effect of hydrogen peroxide on the growth of the three strains, we performed a strain growth test (Fig. 7). At a selected sub-MIC concentration of hydrogen peroxide (0.1%), the growth of strain D2 is much slower compared with both wild-type and strain C2. At 36 h post exposure, the OD600 of strain D2 is at around 0.3, while are at around 0.9 and 1.2 of wild-type strain and strain C2, and the difference is statistical significant (*p* < 0.05). Similar trend was presented at 48 h post exposure, in which the difference of OD600 between strain D2 and WT/strain C2 is statistical significant (*p* < 0.05). We concluded that Ste2 has a protective role against oxidative stress resulted from hydrogen peroxide, while the detailed mechanism will require future investigation.

## Discussion

Bacterial EPSs are sources of medically important substances: many microbial EPSs are found to have anti-inflammation, anti-tumor, anti-aging and anti-rheumatic properties. They are synthesized via biosynthetic enzymes, being secreted into the surrounding environment, and are often important for the biofilm formation and pathogenicity [31]. As for the function of EPSs to their producing organisms, the regulation of oxidative stress has been linked to EPS synthesis in *Streptococcus mutans* [32]. The deletion of the diadenylate cyclase CdaA encoding gene causes decreased c-di-AMP levels, increased sensitivity to hydrogen peroxide and increased production of EPSs in *S. mutans* [32]. The function of *Streptomycetes*-origin EPSs has not been well studied yet: besides from Ebosin from *Streptomyces* sp. 139, only a few other *Streptomyces*-origin EPSs have been reported, i.e., poly-β-1,6-N-acetylglucosamine from *Streptomyces coelicolor* and *Streptomyces lividans* possibly participates in the attachment of bacteria to hydrophilic surfaces [33].

DasRABC (Deficient in aerial mycelium and spore formation) was first identified in *S. griseus* as an ATP-binding cassette transport system involved in regulation of morphological differentiation in response to glucose [16]. Overexpression of *dasA* caused ectopic septation in very young substrate hyphae after only one day of growth and subsequent sporulation in response to glucose, while disruption of *dasA* or *dasR* resulted in growth as substrate mycelium [16]. In a later study, DasA has been demonstrated to act as a link between chitin utilization and morphogenesis in *S. coelicolor* [34]. The interruption of *dasA* resulted in an extraordinary and unique phenotype, i.e., spore chains showed extensive germination. The premature gemination could possibly be linked to the disorder of GlcNAc utilization in *dasA* mutants [34]. Since then, *dasRABC* has been extensively studied and has been linked to the Streptomycetes antibiotics biosynthesis as well as aminosugars utilization. For example, DasR is able to bind directly to the promoters of all genes encoding pathway-specific regulators of each all known antibiotics produced by *S. coelicolor*, including undecylprodigiosin, actinorhodin, cpk-cryptic polyketide and cda-calcium dependent antibiotic [35]. Moreover, DasR could sense the nutritional signals and regulate the biosynthesis of secondary metabolites [35, 36]. The *dasABC* gene cluster has been demonstrated to encode an ABC transporter for the uptake of N, N'-Diacetylchitobiose ((GlcNAc)_2_) in *S. coelicolor* A3(2) [37]. Notably, DasA protein had the highest affinity for (GlcNAc)_2_, and the disruption of *dasA* resulted in a four-fold decrease in the uptake rate of (GlcNAc)_2_. GlcNAc and (GlcNAc)_2_ serve as both carbon and nitrogen sources, and are the preferred nutrient sources for Streptomycetes [38]. In this study, *ste2*, which is homologous to *dasA*, has also been demonstrated to play an important role in the utilization of carbon/nitrogen sources including GlcNAc and (GlcNAc)_2_ in *Streptomyces* sp. 139.

Our previous study has linked Ebosin production to Ste1 (GntR family regulator DasR) and demonstrated that Ste1 serves as a transcription repressor during Ebosin biosynthesis by binding to the promoter 1 and 3 regions in the *ste* gene cluster with high affinity [39]. In this study, we focused on Ste2 and found Ste2 to be the negative regulator during Ebosin biosynthesis. The disruption of *ste2* results in a two-fold increase in Ebosin production. Real-time qPCR reveals that genes within Ebosin biosynthetic gene cluster are significantly upregulated in the *ste2* disruption strain. RNA sequencing analysis suggests that the disruption of *ste2* possibly results in the increase of sensitivity to oxidative stress as well as the fixation of carbon and nitrogen sources, and the increased level of Ebosin being secreted would in turn help the bacterial strain to avoid cellular damage resulted from oxidative stress. Transcriptomic analysis reveals that the disruption of *ste2* results in the increase of sensitivity to oxidative stress, which is confirmed by a hydrogen peroxide sensitivity test. The *ste2* disruption strain has a defect in aminosugars uptake as well as has a different morphology compared with the native strain. A list of genes has been found to be affected by the disruption of *ste2*, which will be of future interest to study EPSs biosynthesis and the function of EPSs to its producing strain. To the best of our knowledge, this is the first study which links the *Streptomyces*-origin EPSs production to the cellular morphology, nutrient utilization, and cellular response to oxidative stress.

## Conclusion

Our findings revealed that the sugar binding protein Ste2 controls Ebosin production, aminosugars utilization, sensitivity to oxidative stress, and morphological differentiation of *Streptomyces* sp. 139. Our work points out the possible connection between environmental stress sensing, nutrient uptake and EPSs biosynthesis, thus will be useful for future work aimed at understanding the function of EPSs to their producing organisms and illustrating the biosynthesis of microbial EPSs that are of medical importance.

## Materials and methods

### Bacterial strains, plasmids, primers and growth conditions

Bacterial strains, plasmids, and primers used in this work are listed in Table 2. *E. coli* DH5α was routinely used for vectors construction. All primers were provided by Invitrogen (Beijing, China). *Streptomyces* sp. 139 and mutant strains were cultured in solid minimal (10 mM K_2_HPO_4_, 10 mM KH_2_PO_4_, 1 mM CaCl_2_, 0.5 mM MgCl_2_ supplemented with 0.1% v/v trace element solution) or R2YE medium [40] plus agar (1.5% w/v), and were incubated at 28°C with shaking (250 rpm) either in Tryptic Soy Broth (TSB, USA) supplemented with 5 mM MgCl_2_ and 0.5% glycine or in fermentation medium (1% glucose, 2% starch, 2% soybean extract, 0.2% tryptone, 0.2% beef extract, 0.4% yeast extract, 0.05% K_2_HPO_4_, 0.3% CaCO_3_, pH 7.3). *E. coli* strains were grown aerobically at 37°C in Luria–Bertani (LB) medium or LB solidified with agar (1.5% w/v) supplemented with the appropriate antibiotics (100 μg/mL ampicillin, 100 μg/mL kanamycin or 50 μg/mL apramycin).

**Table 2:** Strains, plasmids, and primers used in this study

Strains, plasmids, and primers	Description	Reference
Bacterial strains		
* Streptomyces* sp. 139	Ebosin producing strain	Our lab
* Streptomyces* sp. 139 D2	*ste2* knockout mutant of *Streptomyces* sp.139	This study
* Streptomyces* sp. 139 C2	*ste2*-complemented strain	This study
* E. coli* DH5α	F^−^ *recA1 endA1 hsdR17 deoR thi-1 supE44 gyrA96 relA1 Δ(lacZYA- argF) U169λ*^*−*^*(φ80dlacZΔM15)*	
* E. coli* ET12567	methylation-deficient *E. coli*; *dam*^*−*^* dcm*^*−*^* hsdM*	44
* E. coli* BL21 (DE3)	F^−^ *ompT hsdSB(r*_*B*_^*−*^* m*_*B*_^*−*^*) dcm gal λ(DE3)*	Novagen
Plasmids		
pKC1139	Shuttle plasmid (*E.coli–Streptomyces)*; pSG5, pBR322; *aac[3]IV lacZa oriT*_*RK2*_; Am^r^	43
pKC2D	pKC1139 derived plasmid carrying F1, F2 and Km^r^ fragments; Km^r^ Am^r^	This study
pKC2C	pKC1139 derived plasmid carrying 0.45-kb ErmE^*^ promoter fragment and *ste2*; Am^r^	This study
pGEM-3Zf-ErmE^*^	Resource of ErmE^*^ promoter; Ap^r^	45
pET30a	T7 promoter, His-tag; Km^r^	Novagen
pET30a-*ste2*	pET30a derived plasmid carrying *ste2*; Km^r^	This study
Primers		
P1 (*Eco*RI)	CTGGAATTCGTGCCCTTGCCCTGGAT	F1
P2 (*Xba*I)	GCCTCTAGACTTCGCCTTGGTCTTCT	F1
P3 (*Xba*I)	GCATCTAGAGGCAGCCAGAAGCAGGAAC	F2
P4 (*Hin*dIII)	AGCAAGCTTACAGGATGGAGCGGAGG	F2
P5 (*Bam*HI)	CGCGGATCCATGGGTGCGCAAGGCATT	*ste2*
P6 (*Hin*dIII)	CCCAAGCTTTTACTGCTGCTGCGCCAG	*ste2*
* ste5* forward	GCTGATCCTGCTGGTGGTGC	
* ste5* reverse	CCATCGTGCGGAACTTGAGG	
* ste8* forward	CTCGGCAAGCTCAGCCAGAC	
* ste8* reverse	CGAGCAGCAGGAACAGCACC	
* ste17* forward	CTGGACGGCGACGAGAT	
* ste17* reverse	CGACGCAGTGGAACGAG	
* hrdB* forward	TGGTCGAGGTCATCAACAAG	
* hrdB* reverse	TGGACCTCGATGACCTTCTC	

### General DNA manipulation

Isolation of *E. coli* plasmid DNA and standard recombinant DNA techniques were performed as previously described [41]. *Streptomyces* plasmid and genomic DNA was isolated as previously mentioned [40].

### Disruption of ste2 in *Streptomyces* sp. 139

Primers are listed in Table 2. Using *Streptomyces* sp. 139 chromosome as template, the 829-bp upstream region of *ste2* (designated F1) was amplified with primers P1 and P2, while the 749-bp downstream region of *ste2* (designated F2) was amplified with primers P3 and P4. P1 contains an *Eco*RI restriction site, P2 contains an *Xba*I restriction site, P3 contains an *Xba*I restriction site and P4 contains a *Hin*dIII restriction site. The PCR protocol is as following: initial denaturation at 98℃ for 3 min, 30 cycles of 20 s at 98℃, 30 s at 62℃, 1 min at 72℃ and finally 10 min at 72℃. A 1.2-kb fragment containing the kanamycin resistance gene (designated F3) was obtained by digesting DNA of *Streptomyces griseus* SS-1198PR with *Xba*I [42].

After ligating the three DNA fragments (F1, F3, then F2), the resulting 3-kb fragment was inserted into the *Eco*RI-*Hin*dIII sites of pKC1139 [43] to create the *ste2*-disruption vector pKC2D. After propagation in *E. coli* ET12567 [44], pKC2D was introduced into *Streptomyces* sp. 139 by polyethylene glycol (PEG)-mediated protoplast transformation [40]. Briefly, plates were incubated at 28℃ for 20 h, before being overlaid with soft R2YE (0.7% agar) containing 40 μg/L of kanamycin. pKC2D bears a temperature-sensitive *Streptomyces* replication origin which is unable to replicate when temperature reaches 34℃. The transformants were first incubated at 28℃ for two days until pinpoint-size colonies appeared, and were shifted to 37 ℃ for further incubation. Mutants resulted from the double-crossover homologous recombination grew out of the original pinpoint-size colonies within a few days. The disruption of *ste2* on the chromosome was confirmed by Southern blot. For Southern blot analysis, a DIG high prime DNA labeling and detection starter kit II (Roche, USA) was used following the manufacturer’s instructions. The resulting *ste2* disruption mutant was designated as strain D2.

### Complementation of *ste2* disruption strain

Using *Streptomyces* sp. 139 chromosomal DNA as template, *ste2* was amplified with primers P5 and P6. Digested with *EcoR*I and *Bam*HI, a 0.45-kb fragment of *erm*E^*^ promoter was isolated from plasmid pGEM-3zf-*erm*E^*^ [45]. Fragment *erm*E^*^ and *ste2* were ligated together and inserted into the *Bam*HI–*Hin*dIII-cut pKC1139 vector to yield pKC2C. After being propagated in *E. coli* ET12567, pKC2C was isolated and transformed into the protoplasts of strain D2. The complementing strain was designated as strain C2.

### Isolation and activity analysis of Ebosin

Ebosin was isolated from a total of 1 L of the supernatant of the fermentation culture of *Streptomyces* sp. 139, Strain D2 and Strain C2 at 28 ℃ for 96 h in triplicate, as previously described [46].

An enzyme-linked immunosorbent assay (ELISA) was used to analyze the binding activity of isolated Ebosin with interleukin-1 (IL-1) for IL-1R [46].

### Determination of glucose, GlcNAc, and (GlcNAc)_2_ concentrations.

To investigate the responses of the cells to sugars, *Streptomyces* sp. 139 and its mutant strain *Streptomyces* sp. 139 D2 were cultivated by following a method described previously [39], with some modifications. Spores of *Streptomyces* sp. 139 strains formed on agar medium were inoculated into 100 mL TSB medium in a 500-mL baffled Erlenmeyer flask and grown for 48 h at 28 °C on a rotary shaker at 200 rpm. Mycelia were harvested by centrifugation (3,000 rpm; 10 min), washed with MM (A minimal medium (MM) (10 mM K_2_HPO_4_, 10 mM KH_2_PO_4_, 1 mM CaCl_2_, 0.5 mM MgCl_2_ supplemented with 0.1% (vol/vol) trace element solution) without carbon sources, suspended in 25 ml MM, and divided into several aliquots(0.8 g/25 ml). Each aliquot was supplemented with various carbon sources, i.e., 250 μM of Glc, GlcNAc, (GlcNAc)_2_ (TCI, Japan). The culture was further grown at 28 °C on a rotary shaker at 200 rpm. Portions of the culture fluids were sampled periodically, centrifuged to separate the supernatants and the mycelia, and stored at -80 °C. The supernatants were subjected to measurements of sugar (Glc, GlcNAc, (GlcNAc)_2_) concentrations, whereas the mycelia were used for total RNA preparation.

### RT-qPCR analysis

*Streptomyces* sp. 139, mutant strain D2 and the complemented strain C2 were cultured at 28 ℃ for 48 h in TSB. 10 mL of the culture were inoculated into 50 mL fresh TSB and were cultured at 28 ℃ for 24 h, 48 h, and 96 h, respectively. At each time point, the mycelia of the three strains were collected by centrifugation (3,000 g; 10 min) and washed with PBS (NaCl 137 mM, KCl 2.7 mM, Na_2_HPO_4_ 10 mM, KH_2_PO_4_ 2 mM, pH 7.4), respectively. Total RNA of each mycelia sample was isolated with the TRIzol System (TransGen) according to the manufacturer’s instructions. Remaining DNA was removed by DNAse (TaKaRa). cDNA was synthesized with Superscript III First-Strain synthesis system kit for RT-PCR (TransGen) according to the manufacturer’s instruction. Primers for each gene are listed in Table 1. 50 ng cDNA was used for each qPCR reaction with FastStart Universal SYBR Green Master kit (Roche) following the manufacturer’s instructions. All transcripts were normalized according to *hrd*B (RNA polymerase principal sigma factor) transcript quantities.

### Microscopy

Surface-grown aerial hyphae and spores of *Streptomyces* sp.139, Strain D2 and strain C2 were examined by scanning electron microscopy (SEM) after four days of growth on minimal and R2YE agar, respectively. The agar blocks containing spores and hyphae were cut and fixed in 2.5% glutaraldehyde in 0.1 M phosphate buffer pH 7.0 for overnight at 4 °C, washed three times in 0.1 M phosphate, post-fixed in 1% osmium tetroxyde, 2–4 h in 0.1 M phosphate, then 15–20 min in ethanol 30%, 50%, 70%, 85%, 95% and 100%, respectively. Each specimen was rinsed, dehydrated, coated with platinum-gold and examined with a FEI QUANTA 200 scanning electron microscope [47].

### RNA sequencing

*Steptomyces* sp. 139 and its mutant Strain D2 was cultured in 10 mL TSB medium at 28℃ for 36 h in triplicate. The culture was inoculated 1:10 (vol/vol) into 50 ml fresh TSB medium and incubated at 28 ℃ for 24 h. After centrifugation at 5,000 rpm for 10 min, the cell pellet was harvested, flash frozen in liquid nitrogen, and sent out for RNA extraction and RNA sequencing (Beijing Genomics Institute, Shenzhen, China). RNA sequencing data have been deposited in NCBI SRAs under the BioProject accession PRJNA647684 and can be accessed with the following link: https://www.ncbi.nlm.nih.gov/bioproject/PRJNA647684.

### H_2_O_2_ sensitivity experiments

A minimum inhibition concentration (MIC) test of H_2_O_2_ against *Streptomyces* sp. 139 wild-type, D2 and C2 strain was performed in TSB according to the CLSI protocol [48]. The MIC of H_2_O_2_ against wild-type, D2 and C2 strain was 1%, 0.25% and 1%, respectively. The effect of H_2_O_2_ on the growth of strains was tested at a sub-MIC concentration (0.1%). The initial concentration of strains was adjusted to OD600 0.1. Bacterial strains were cultured at 28℃ with shaking and their growth was monitored with an 800TS microplate reader (BioTek Instruments Inc.) at 0 h, 1 h, 2 h, 4 h, 8 h, 16 h, 24 h, 36 h and 48 h. Experiments were done at least in triplicate.

## Supplementary Information

**Additional file 1.**

**Additional file 2.**

## Data Availability

All documents and additional data are available from the corresponding author upon reasonable request. RNA sequencing data have been submitted to the Sequence Read Archive database of National Center for Biotechnology Information under the accession PRJNA647684 and can be accessed with the following link: https://www.ncbi.nlm.nih.gov/bioproject/PRJNA647684.
