# Supplementary material for: A DasA family sugar binding protein Ste2 links nutrient and oxidative stress to exopolysaccharides production in Streptomyces sp. 139
Source: BMC Microbiol. 2022 Mar 8;22:69. doi: 10.1186/s12866-022-02472-7 (PMC8902715; doi:10.1186/s12866-022-02472-7)
Supplement: Supplementary file 1 — Additional file 1. [file 12866_2022_2472_MOESM1_ESM.docx]

**A DasA family sugar binding protein Ste2 links nutrient and oxidative stress to exopolysaccharides production in *Streptomyces* sp. 139**

Mengxin Geng^1^, Limei Ai^1^, Ming Ma^1^, Panpan Li^2^, Lianhong Guo^1^, Guangzhi Shan^2^, Liping Bai^1*^

^1^ NHC Key Laboratory of Biotechnology of Antibiotics, CAMS Key Laboratory of Synthetic Biology for Drug Innovation, Institute of Medicinal Biotechnology, Chinese Academy of Medical Sciences & Peking Union Medical College, Beijing 100050, China

^2^ Analytical & Testing Center, Institute of Medicinal Biotechnology, Chinese Academy of Medical Sciences & Peking Union Medical College, Beijing 100050, China

*** Correspondence:**Corresponding Author
[lipingbai1973@163.com](mailto:lipingbai1973@163.com)

Tel./Fax: (010)63013336

**Supplementary Figure 1.** **Analysis of *ste234* gene.** (A) Amino acid alignment of Ste2 (AAN04229) with sugar binding protein DasA. DasA from *Streptomyces coelicolor* A3 (2) (NP_629379), and *Streptomyces griseus* (BAB79297) are shown. (B) Amino acid alignment of Ste3 (AAN04230) with integral membrane protein DasB. DasB from from *Streptomyces coelicolor* A3 (2) (NP_629380), and *Streptomyces griseus* (BAG19109) are shown. (C) Amino acid alignment of Ste4 (AAN04231) with integral membrane protein DasC. DasC from *Streptomyces coelicolor* A3 (2) (NP_629381), and *Streptomyces griseus* (BAG19108) are shown. Note: B and C have been previously published by our group (Bai et al., 2015).

A.


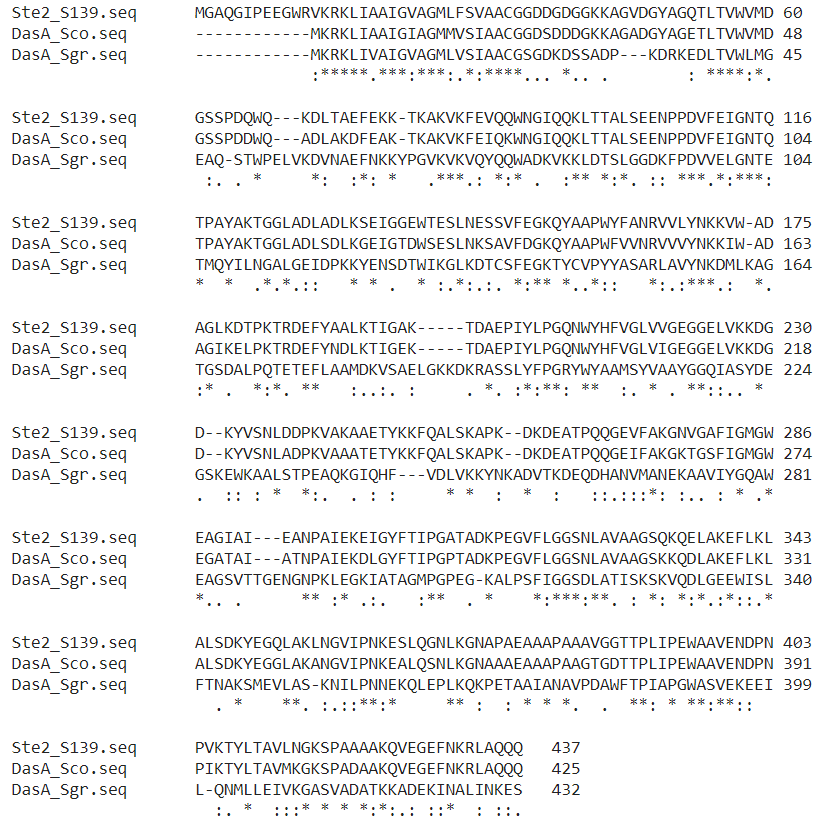


B.


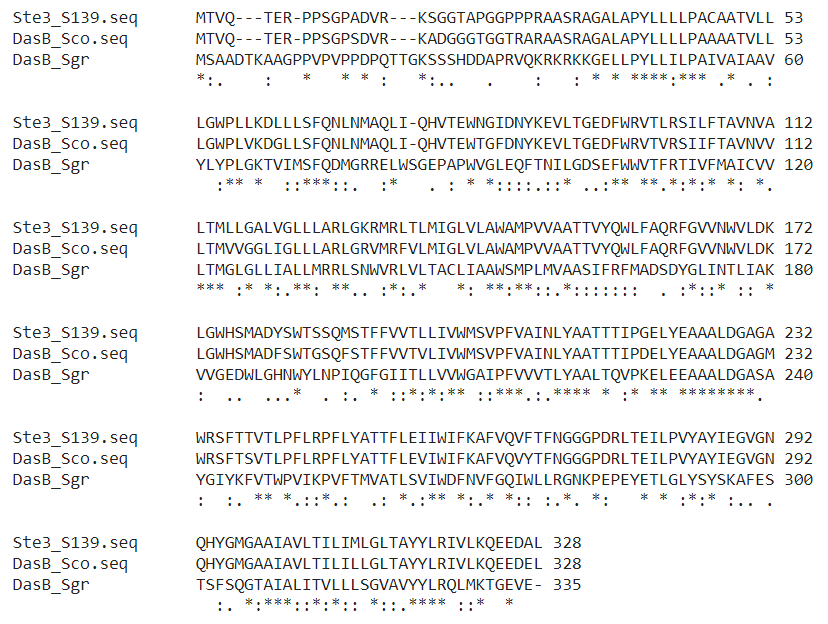


C.


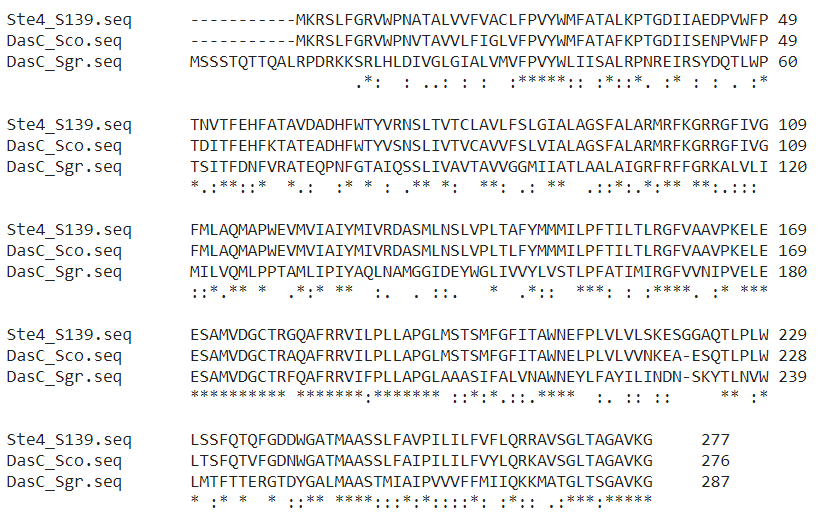


**Supplementary Figure 2 Southern blotting analyses of the wild-type *Streptomyces* sp. 139 and Strain D2 (full length image of Figure 1B).** (Line 2) *Streptomyces* sp. 139 chromosome DNA digested with *Bam*HI and *Hin*dIII, (Line 3) Strain D2 chromosome DNA digested with BamHI. (M) DNA marker. A DIG-labeled F1 served as the hybridization probe. Note: Line 1, 4, and 5 were intended to demonstrate other questions and were not related to the current manuscript.


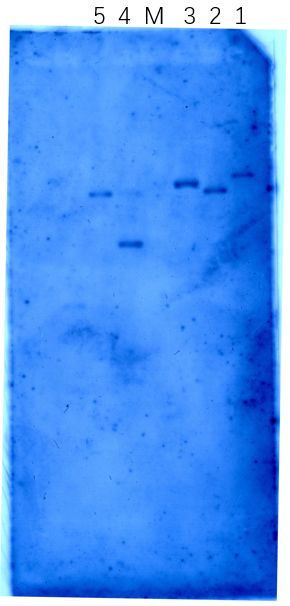


**References**

Bai L, Jiang R, Guo L, Zhang Y, Li Y. 2015. The Effects of *ste3* and *ste4* Genes Double Disruption in Ebosin Biosynthesis. China Biotechnol. 35: 23-28.
